# Comparison of reduced field-of-view DWI and full field-of view DWI for the differentiation between non-muscle invasive bladder cancer and muscle invasive bladder cancer using VI-RADS

**DOI:** 10.1371/journal.pone.0271470

**Published:** 2022-07-20

**Authors:** Hiroshi Juri, Akira Higashiyama, Kiyohito Yamamoto, Yoshifumi Narumi, Haruhito Azuma, Kazuhiro Yamamoto, Keigo Osuga

**Affiliations:** 1 Department of Diagnostic Radiology, Faculty of Medicine, Osaka Medical and Pharmaceutical University, Takatsuki, Osaka, Japan; 2 Department of Health Care, Osaka Medical Association Center, Osaka, Japan; 3 Department of Urology, Faculty of Medicine, Osaka Medical and Pharmaceutical University, Takatsuki, Osaka, Japan; Public Library of Science, UNITED STATES

## Abstract

**Purpose:**

To evaluate whether reduced field-of-view (rFOV) DWI sequence improves the differentiation between non-muscle-invasive bladder cancer (NMIBC) and muscle-invasive bladder cancer (MIBC) using VI-RADS.

**Material and methods:**

Eighty-nine patients underwent bladder MRI with full field-of-view (fFOV) DWI and rFOV DWI sequence. Images were independently evaluated by 2 radiologists. The sensitivities, specificities, accuracies, and areas under the curve (AUCs) for the differentiation between NMIBC and MIBC with fFOV DWI and with rFOV DWI sequence were calculated using VI-RADS. Apparent diffusion coefficients (ADC) values were measured for each patient and averaged.

**Results:**

The sensitivity, specificity, accuracy, and AUC by reader 1 were 92%, 78%, 82% and 0.905 with fFOV DWI, and 92%, 86%, 88% and 0.916 with rFOV DWI sequence, respectively. The sensitivity, specificity, accuracy and AUC by reader 2 were 96%, 76%, 82% and 0.900 with conventional DWI, and 96%, 81%, 85% and 0.907 with rFOV DWI sequence, respectively. The specificity and accuracy of reader 1 were significantly better with rFOV DWI sequence than with fFOV DWI, in contrast there was no significant difference for the others. The average of ADC values of fFOV DWI and rFOV DWI sequence were 1.004×10^−6^ mm^2^/s and 1.003×10^−6^ mm^2^/s, respectively.

**Conclusion:**

The diagnostic ability of rFOV DWI sequence may be better than that of fFOV DWI using VI-RADS for the differentiation between NMIBC and MIBC regardless of image-reading experience, it is controversial.

## Introduction

Bladder cancer is one of the most common cancers encountered by urologists. Distinction between non-muscle-invasive bladder cancer (NMIBC) and muscle-invasive bladder cancer (MIBC) is critical for treatment planning. Magnetic resonance imaging (MRI) and CT are both important modalities for staging of bladder cancer. CT is generally used to evaluate extravesical invasion of bladder cancer while multiparametric-MRI (mp-MRI), used to distinguish between NMIBC and MIBC, has become the most important imaging technique [1]. Meta-analyses of mp-MRI data were published in 2017–18 [2, 3], and the Vesical Imaging Reporting and Data System (VI-RADS) was released in 2018 to standardize the scanning and reporting criteria based on mp-MRI [4].

Diffusion-weighted imaging (DWI) is one of the routine protocols of mp-MRI for bladder cancer and is the most commonly used sequence for determining the VI-RADS score [4, 5]. Currently, full field-of-view (fFOV) single-shot echo-planar imaging is the standard sequence used for conventional DWI. However, the spatial resolution of this technique is not sufficient, as it is limited by artifacts of magnetic susceptibility. Recently, a reduced field-of-view (rFOV) sequence has been obtained to reduce artifacts in DWI [6–8]. Zoomed DWI is one of the rFOV sequences provided by Philips Healthcare. In Zoom DWI, the 180° refocusing pulse is applied obliquely to the 90° slice excitation pulse. In addition, outer volume suppression (OVS) is applied. As a result, the field of view (FOV) is reduced, and artifacts due to peristalsis of the bowel tract may be reduced [8]. With Zoomed DWI, the ability to discriminate NMIBC and MIBC may be better than with conventional DWI. Recently, a few reports of rFOV sequence for bladder cancer have been published [9–11]. However, there have been few reports evaluating the diagnostic ability of the differentiation between NMIBC and MIBC of bladder cancer on rFOV DWI sequence using VI-RADS.

The aim of this study was to evaluate whether rFOV DWI improves the differentiation between NMIBC and MIBC of bladder cancer using VI-RADS.

## Materials and methods

### Patients

**Fig 1:**
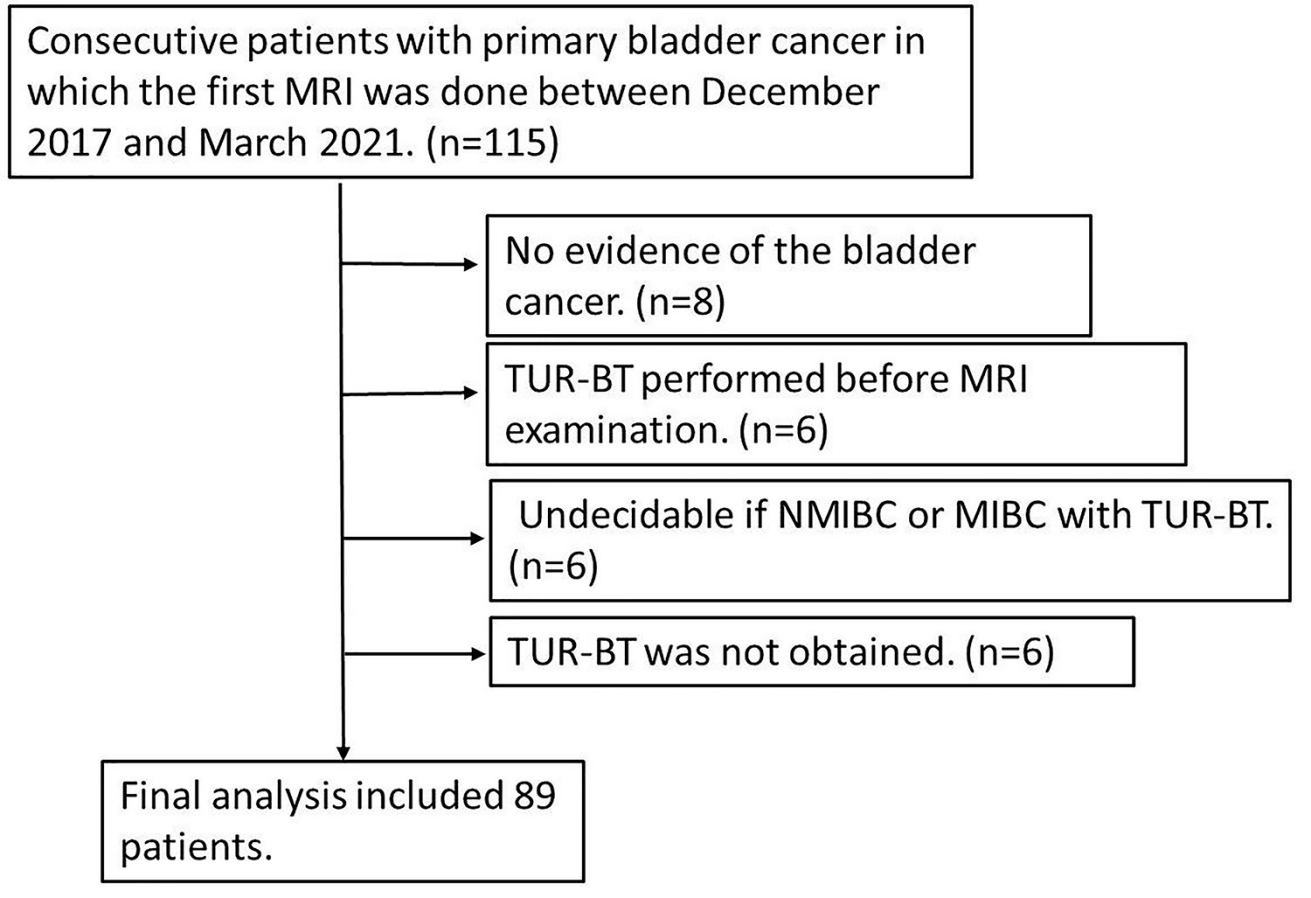
Flow diagram of the selection of patients.

This study was approved by the institutional review board (IRB) of Osaka Medical and Pharmaceutical University, and written informed consent was waived because of the retrospective design. One hundred fifteen consecutive patients with clinically suspected bladder cancer between December 2017 and March 2021 were enrolled. The exclusion criteria were as follows: (1) no evidence of bladder cancer; (2) transurethral resection of the bladder tumor (TUR-BT) performed before MRI examination; (3) ambiguity as to whether a case is NMIBC or MIBC based on transurethral biopsy; or (4) TUR-BT was not performed. Eighty-nine patients were included in the evaluation (70 men and 19 women; age 37–81 years (mean, 69.7 years) (Fig 1).

### MRI technique

All MRI examinations were performed on a 3.0-T MR scanner (Ingenia 3.0T, Phillips Healthcare). To moderately distend the urinary bladder, patients were prohibited from urinating for at least 1 hour before examination. The imaging protocols included axial T2-weighted imaging (T2WI) using a turbo spin-echo sequence (TSE), three-dimensional T2-weighted volumetric interpolated examination (3D VISTA), axial fFOV DWI, axial Zoomed DWI, and dynamic contrast-enhanced imaging (DCE) with three-dimensional T1-weighted volumetric interpolated examination (3D T1WI eTHRIVE). In patients with contraindications to contrast media or severe renal failure, DCE was omitted. The MRI protocol is listed in Table 1.

**Table 1:** MRI protocol of the bladder MRI.

	T2WI (2D TSE)	T2WI (3D VISTA)	fFOV DWI	Zoomed DWI	DCE T1WI (3D eTHRIVE)
Direction	Axial	Axial	Axial	Axial	Axial
TR (ms)	4000~8000	1600	6300	3000	3.4
TE (ms)	90	180	75	75	1.7
Band width (Hz)	218.2	555.6 (Hz)	28.8	24.7	723.4
b value (s/mm^2^)	−	−	1000	1000	−
Matrix	320×224	304×304	128×128	96×96	240×190
FOV (mm)	240×240	300×300	330×288.7	200×115.6	240×240
Slice thickness / Gap (mm)	3/1	1.4/0.7	2.5/ 0	3/0	1.4/0.7
NSA	1	1	9	8	1
Scan time (mm:ss)	2:37	5:33	4:48	5:24	0:20 (×5)

### Qualitative analysis

Images were analyzed using the Picture Archiving and Communication Systems (PACS) and were independently evaluated by 2 radiologists with 4 (reader 1) and 8 (reader 2) years of experience for bladder MRI, respectively. We evaluated the MRI images of consecutive all patients in this study. Per-patient analyses were performed in this study. In cases with multiple lesions, the highest tumor load in each patient was selected for evaluation.

First, each patient’s images from conventional DWI and Zoomed DWI were scored with a 3-point quality score based on the sharpness, artifacts and overall image quality of the urinary bladder: 1 = poor, affecting the diagnosis, with no sharp margin or severe artifacts, lesions unidentifiable; 2 = normal, with moderately sharp margins or mild artifacts, lesion identifiable; 3 = good, with sharp margins or no artifacts, lesion clearly identifiable. The average scores of each item of image quality for conventional DWI and Zoomed DWI were calculated.

**Fig 2:**
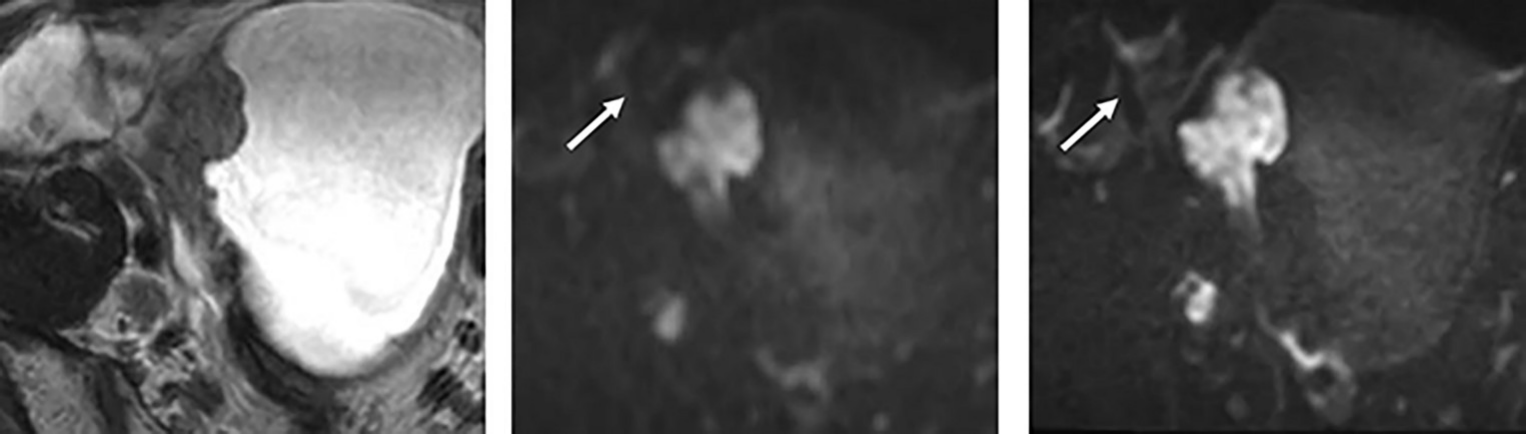
53-year-old man with the urothelial carcinoma of pT2 or more on the right side wall of the bladder. (A) T2WI (B) fFOV DWI (C) Zoomed DWI. The bladder wall demonstrated more clearly on Zoomed DWI than on fFOV DWI. An artifact appears from the intestine and the rectum in both images of fFOV and Zoomed DWI. VI-RADS score was 5 for both readers.

Second, the differentiation between NMIBC and MIBC of bladder cancer was performed. Each radiologist reviewed 3 image sets: T2WI (the axial T2WI FSE and the 3D VISTA), DWI (conventional DWI or Zoomed DWI), and DCE. In this analysis, we evaluated each lesion with the total VI-RADS score (Fig 2; 1 = muscle invasion is highly unlikely, 2 = muscle invasion is unlikely, 3 = equivocal, 4 = muscle invasion is likely, and 5 = muscle invasion is highly likely) using T2WI, conventional DWI and DCE, or using T2WI, Zoomed DWI and DCE with an interval of 2 weeks.

T2WI was used as the first-pass imaging, to understand the anatomical relations of the muscle and tumor layers. The commonly used sequence for determining the VI-RADS score was DWI (first) and DCE (second) [4]. DWI was used for the final score when DWI was available. DCE was used for the final scoring when the DWI results were suboptimal. If DCE was also suboptimal, T2WI was used for the final score. If the lesion was very small (<1cm), we gave it a score of 1. A score of ≧3 was considered a positive finding of MIBC [12–15].

The results of TUR-BT were the standard references for the differentiation between NMIBC and MIBC of bladder cancer, and stage T2 or more on the pathological result of TUR-BT was considered a positive finding of MIBC. The 5-point scores using VI-RADS were compared with the pathological results of TUR-BT.

### Quantitative analysis

To measure the apparent diffusion coefficient (ADC) values, freehand ROIs were manually drawn on the main lesion of bladder cancer on the ADC maps of fFOV DWI and Zoomed DWI in each patient. We measured ADC values using single slice of ADC map. If the lesion contained a tumor stalk or thickened submucosa, ROIs were drawn to exclude these structures because they have no tumor component [16]. The average values of the ADC values for conventional DWI and Zoomed DWI were calculated.

### Statistical analysis

Statistical analyses were performed with JMP pro 15.0. Comparison of scores of each item of image quality was subsequently performed using the Wilcoxon signed-rank test. To evaluate the sensitivity, specificity and accuracy of T staging, we compared results with McNemar’s test. Using 5-scale scores, receiver operating characteristic (ROC) curves were drawn, and observer performance for T staging in each sequence was estimated by calculating the area under the curve (AUC). Differences between the AUCs were estimated. Comparisons of ADC values were subsequently performed using Student’s t test. *P*-values <0.05 were considered to indicate a significant difference.

We used the kappa statistic to evaluate the agreement of the 2 reviewers for T staging. A κ of <0.20 was considered poor; 0.21–0.40, fair; 0.41–0.60, moderate; 0.61–0.80, good; and 0.81–1.00, excellent.

## Results

MRI scans of these patients were conducted 2 to 70 days (mean 26 days) before TUR-BT. The number of patients with NMIBC and MIBC were respectively 63 and 26 according to the pathological results of TUR-BT.

The results of image quality analysis for conventional DWI and Zoomed DWI are shown in Table 2.

**Table 2:** Assessment of the image quality.

	fFOV DWI	Zoomed DWI	p
**Reader 1**			
** Sharpness**	1.79±0.40	2.10±0.43	<0.001
** Artifacts**	1.84±0.37	1.79±0.40	0.045
** Overall image quality**	1.92±0.27	2.25±0.51	<0.001
**Reader 2**			
** Sharpness**	1.74±0.47	2.52±0.52	<0.001
** Artifacts**	2.26±0.72	2.22±0.70	0.10
** Overall image quality**	1.61±0.48	2.26±0.57	<0.001

Data are listed as mean±SD.

The scores were significantly better on Zoomed DWI than on conventional DWI in the sharpness and overall quality, but the scores of artifacts were similar on Zoomed DWI and on conventional DWI (p = 0.10) for the reader 2. For reader 1, the scores were significantly better on Zoomed DWI than on conventional DWI in terms of sharpness and overall quality but worse in terms of artifacts (p = 0.045). Examples of images from conventional DWI and Zoomed DWI are shown in Fig 2.

The results of sensitivity, specificity, accuracy and AUC for the differentiation between NMIBC and MIBC are shown in Table 3.

**Table 3:** Diagnostic abilities of T staging of the bladder cancer.

	fFOV DWI	Zoomed DWI	p
**Reader 1**			
** sensitivity**	24/26 (92)	24/26 (92)	1.00
** specificity**	49/63 (78)	54/63 (86)	0.025
**accuracy**	73/89 (82)	78/89 (88)	0.025
**AUC**	0.905	0.916	0.068
**Reader 2**			
** sensitivity**	25/26 (96)	25/26 (96)	1.00
** specificity**	48/63 (76)	51/63 (81)	0.08
**accuracy**	73/89 (82)	76/89 (85)	0.08
**AUC**	0.900	0.907	0.132

Data in parentheses are percentages.

**Fig 3:**
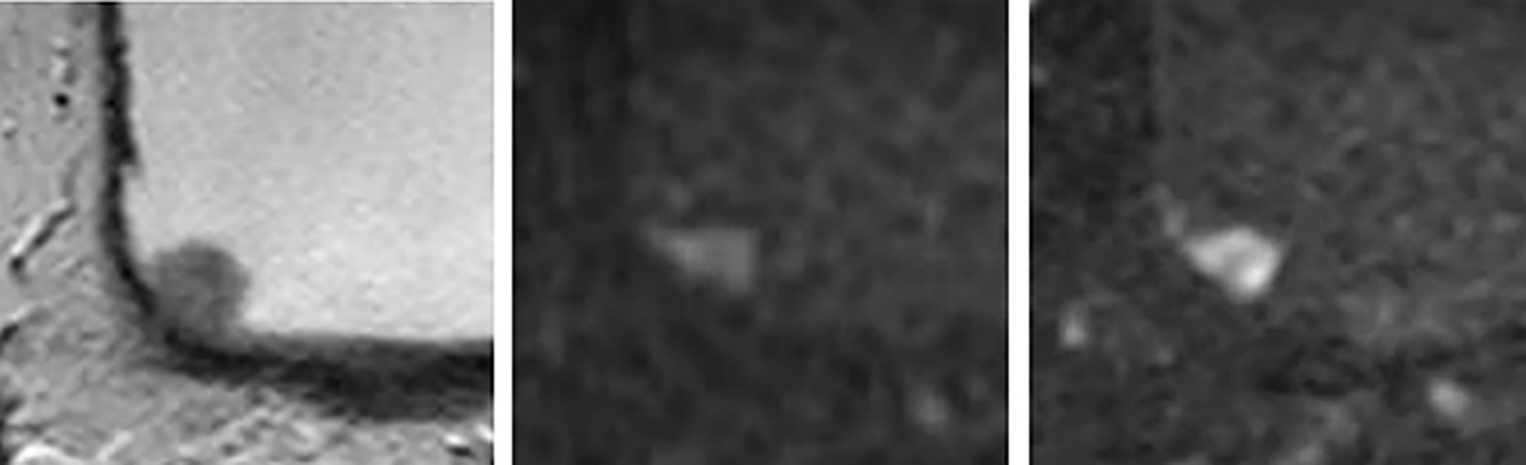
70-year-old man with the urothelial carcinoma of pTa on the right lateral to posterior wall of the bladder. (A) T2WI (B) fFOV DWI (C) Zoomed DWI. The tumor appeared as the lesion without tumor stalk in fFOV DWI, and VI-RADS score was 3 for both readers. In contrast, the tumor appeared as a papillary lesion with tumor stalk in Zoomed DWI, and VI-RADS score was 2 for both readers.

For the reader 1, five lesions received scores of 3 on conventional DWI and 2 on Zoomed DWI (Fig 3).

**Fig 4:**
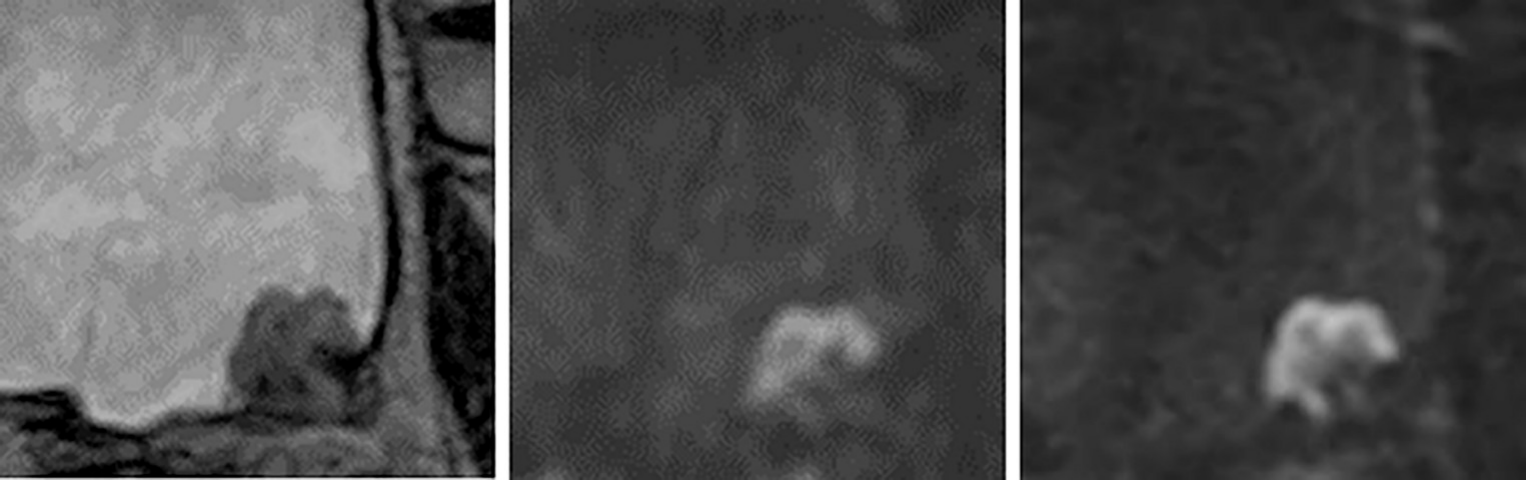
61-year-old woman with the urothelial carcinoma of pTa on the posterior wall of the bladder. (A) T2WI (B) fFOV DWI (C) Zoomed DWI. VI-RADS score was 3 for reader 1 and 2 for reader 2 with fFOV DWI. In contrast, the tumor stalk clearly appeared on Zoomed DWI, and VI-RADS score was 2 for both readers.

For the reader 2, three lesions received scores of 3 on conventional DWI and 2 on Zoomed DWI for reader 2 (Fig 4).

**Fig 5:**
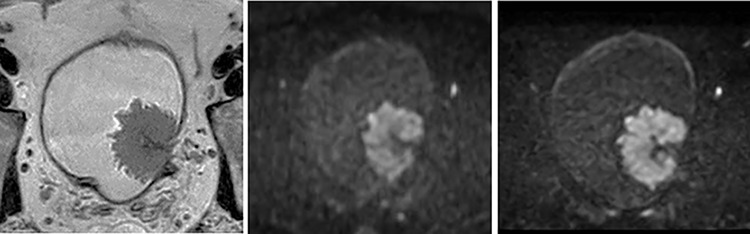
83-year-old woman with the urothelial carcinoma of pT1 on the left lateral wall of the bladder. (A) T2WI (B) fFOV DWI (C) Zoomed DWI. The tumor appeared as a papillary lesion with tumor stalk in both fFOV and Zoomed DWI, and VI-RADS score was 2 for both series.

**Fig 6:**
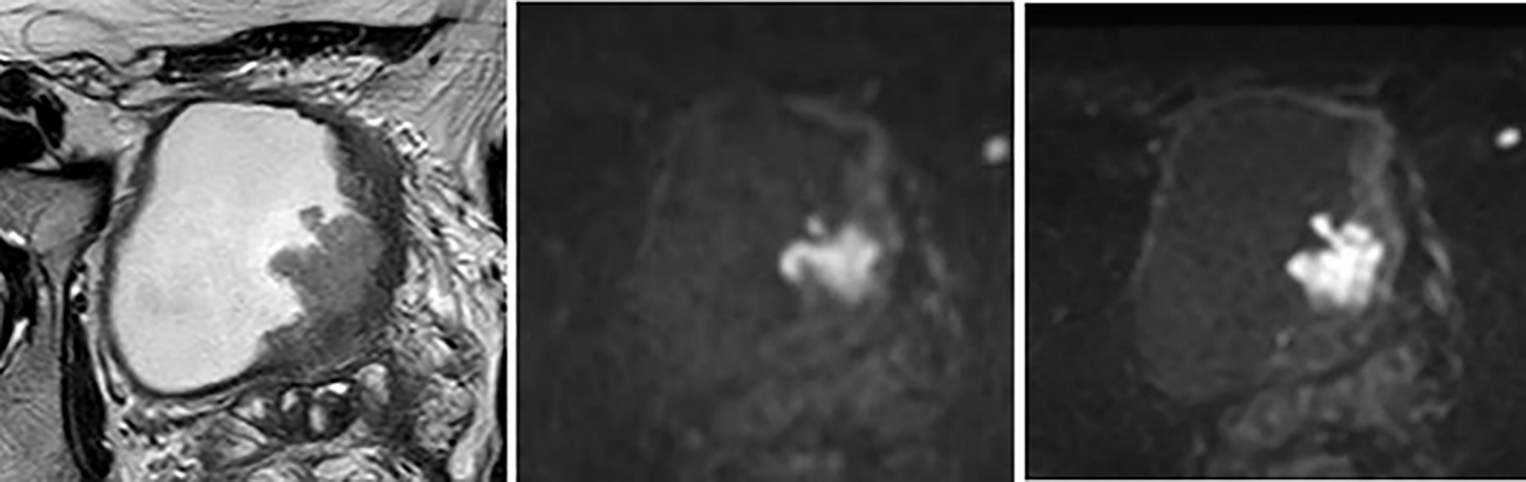
66-year-old man with the urothelial carcinoma of pT2 on the anterior wall of the bladder. (A) T2WI (B) fFOV DWI (C) Zoomed DWI. The tumor appeared as a lesion without stalk in both fFOV and Zoomed DWI, and score of VI-RADS was 3 for both fFOV and Zoomed DWI series.

Evaluation was made with DCE for the final score in 4 patients because the DWI was suboptimal. In 4 patients, both conventional DWI and Zoomed DWI were suboptimal. Only conventional DWI was suboptimal in 2 other patients. In the 2 patients with suboptimal conventional DWI only, the DCE score was the same on Zoomed DWI; thus, the VI-RADS scores were the same for both conventional DWI and Zoomed DWI. The scores of DWI for the differentiation between NMIBC and MIBC in the other patients were the same for both conventional DWI and Zoomed DWI (Figs 5 and 6)

There was no significant difference in sensitivity between the two techniques, either for the reader 1 or the reader 2. The specificity and accuracy on Zoomed DWI were significantly higher than those on conventional DWI for the reader 1 (p = 0.025), but not significantly higher for the reader 2 (p = 0.08). The AUCs were slightly better on Zoomed DWI than on conventional DWI, but there was no significant difference for either the reader 1 or 2 (p = 0.068 and 0.132). The κ values were 0.696 on conventional DWI and 0.701 on Zoomed DWI. Therefore, the interobserver agreement was good for the differentiation between NMIBC and MIBC.

The mean ADC values of main lesions were 1.038 ± 0.21 ×10^−3^ mm^2^/s^　^and 1.023 ±0.20 ×10^-3^mm^2^/s on conventional DWI and Zoomed DWI, respectively. There was no significant difference (p = 0.65).

## Discussion

Zoomed DWI is one of techniques of rFOV DWI sequence. A few reports of rFOV DWI sequence for bladder cancer have been published [9–11], and the good image quality or superiority for diagnosis of the differentiation between NMIBC and MIBC of bladder cancer using rFOV DWI sequence were mentioned in these reports. If the rFOV DWI sequence is better than the conventional DWI using VI-RADS for the distinction of NMIBC and MIBC, the superiority of rFOV DWI sequence might be firmer. Thus, we evaluated Zoomed DWI for the differentiation between NMIBC and MIBC of bladder cancer using VI-RADS.

In qualitative analyses, the image quality was significantly better on Zoomed DWI than on conventional DWI. Especially, the sharpness scores were better on Zoomed DWI than on conventional DWI. The small FOV of Zoomed DWI resulted in a small pixel size, allowing for better sharpness. In contrast, the artifact score was not significantly different between Zoomed DWI and conventional DWI. Even if a small FOV was applied, a segment of colon or small intestine near the urinary bladder could be included in the FOV. The motion artifacts of these structures often affect the bladder wall; thus, a small FOV could not decrease artifacts in these cases. However, overall image quality for the urinary bladder was significantly better on Zoomed DWI than on conventional DWI. The good image quality on Zoomed DWI is due to image sharpness. Our study was similar to previous studies using reduced FOV DWI with a different mechanism [9, 10].

If a score of ≧3 was considered a positive finding of MIBC, the accuracy of the reader 1 was significantly better on Zoomed DWI. The sharpness of the bladder wall was better on Zoomed DWI than on conventional DWI, so results would be better in some cases. Moreover, in these lesions, the low-intensity area of tumor stalk was portrayed more clearly on Zoomed DWI than on conventional DWI in a few lesions, and could be subtracted appropriately. In contrast, the accuracy for the differentiation between NMIBC and MIBC with VI-RADS of the reader 2 was not significantly better on Zoomed DWI than on conventional DWI. Although it may be due to the small number of patients, there may be no significant difference in terms of accuracy in the differentiation between MIBC and NMIBC on the basis of the results of the reader 2. In the clinical course of bladder cancer, the differentiation between NMIBC and MIBC is the most important factor determining treatment plans. A false positive result for MIBC would be problematic as it would lead to an unnecessary deep TUR-BT. Due to the clear delineation of the tumor stalk, Zoomed DWI using VI-RADS may be useful for avoiding overstaging before TUR-BT by a radiologist of any experience level, while maintaining a high sensitivity. Thus, unnecessary deep TUR-BT may be avoided using Zoomed DWI. Moreover, reduction of medical costs may be achieved for some patients if unnecessary second TUR-BT is avoided using evaluation of VI-RADS with Zoomed DWI. Wang Y et al. [11] concluded that the accuracy of T staging and the Az value with reduced FOV DWI are better than those with conventional DWI. However, VI-RADS score system was not used in this study. Thus, our results may be more practical in the future.

There have been several reports for the diagnostic ability of VI-RADS. In some reports, VI-RADS is feasible using the score of 3 as the cutoff value [12–15]. On the other hand, VI-RADS is feasible using the score of 4 as the cutoff value [12, 13, 17, 18]. In the same series, sensitivity is better using the score of 3 as the cutoff value, in contrast specificity is better using the score of 4 as the cutoff value for the distinction between NMIBC and MIBC [12, 13]. In our study, sensitivity was higher than specificity on both groups. It would be due to the cutoff value with the score of ≧3 as the positive finding of MIBC. If we use the score of 4 as the cutoff value, sensitivity might be lower. Moreover, sensitivity, specificity and accuracy would be the same between the two groups. Thus, if the score of 4 is used as the standard cutoff value in the future, there might be no superiority of Zoomed DWI for the distinction between NMIBC and MIBC. However, there are several patients with MIBC in the group of score of 3 for VI-RADS. In addition, the sensitivity for the differentiation between NMIBC and MIBC in our study was over 90% even in the reader 2 with short experience of the bladder MRI. Therefore, we suggest the score of 3 as the cutoff value.

ADC values are not significantly different between conventional DWI and Zoomed DWI. In a previous study, ADC values were significantly different between high- and low-grade bladder cancers [16]. Therefore, ADC values are partially helpful to distinguish the histologic grades of bladder cancer. If ADC values are significantly different between conventional DWI and Zoomed DWI, ADC values of Zoomed DWI cannot be compared to those of conventional DWI in the previous study. Thus, ADC values of Zoomed DWI can be used in the clinical course based on our results.

There are some limitations to our study. First, the degree of distention of the urinary bladder differed across patients in this study. To moderately distend the urinary bladder, patients were prohibited from urinating for at least 1 hour before examination. As a result, there was no patient without sufficient distention of the urinary bladder. Thus, differences in distention did not pose a problem. Second, we did not use an anticholinergic agent before the examination. There are several patients with benign prostatic hyperplasia, and anticholinergic agents are contraindicated in these patients. There would be some artifacts without an anticholinergic agent, but it would be on both conventional DWI and Zoomed DWI because a segment of colon or small intestine near the urinary bladder can be contained even in the small FOV of Zoomed DWI. Third, bi-planar DWI was not acquired in our study. If bi-planar DWIs along the vertical plane of the bladder cancer had been acquired, the diagnostic ability might be better for the differentiation between NMIBC and MIBC on both conventional and Zoomed DWI. In the limited examination time at our institution, bi-planar DWI for both conventional and Zoomed DWI are difficult to obtain. Further prospective studies, with larger population, using bi-planar DWI are needed to validate our initial results.

In conclusion, the image quality of rFOV DWI sequence for bladder cancer is better than that of fFOV DWI. The diagnostic ability of r FOV DWI for the differentiation between MIBC and NMIBC using VI-RADS may be better than that of fFOV DWI regardless of image-reading experience, it is controvercial.
